# Editorial: Innovations in quality of care

**DOI:** 10.3389/frhs.2023.1352322

**Published:** 2024-01-05

**Authors:** João Breda, Ara Darzi, Hutan Ashrafian, Francisco Goiana-da-Silva, Natasha Azzopardi-Muscat

**Affiliations:** ^1^ WHO Athens Quality of Care and Patient Safety Office, World Health Organization Regional Office for Europe, Athens, Greece; ^2^ Institute of Global Health Innovation, Imperial College London, London, United Kingdom; ^3^ National Health System Executive Council, Porto, Portugal; ^4^ Division of Country Health Policies and Systems, World Health Organization Regional Office for Europe, Copenhagen, Denmark

**Keywords:** sustainable development, World Health Organization, quality of care, SARS-CoV-2 pandemic, medical and surgical innovation, Institute Of Global Health Innovation

**Editorial on the Research Topic** Innovations in quality of care

The COVID-19 pandemic has greatly influenced the way we approach health, science, and policy-making (1). This shift has led to more inclusive and effective strategies for change. It has emphasized the importance of better, structured, and opportunity-focused ways of finding and using talent that enhance problem-solving skills and promote creative thinking (2). Globally, there is a shift toward fostering innovation by creating new or revamping old digital and other infrastructures to keep pace with fast-changing societal needs, particularly in delivering essential health services and streamlining organizational management (3).

The European Programme of Work 2020–2025, “United Action for Better Health in Europe”, and the United Nations' Sustainable Development Goals place an emphasis on achieving universal health coverage by ensuring that health services are of sufficient quality to be safe, effective, and accessible (4, 5). This involves meticulous planning, execution, and continuous evaluation (6). In this context, the World Health Organization's Quality of Care and Patient Safety Office in Athens hosted the inaugural “Meeting of the Minds” Conference on quality of care in December 2021. This conference aimed to outline a roadmap for the implementation of key actions in five thematic areas: (1) Innovation and best practices in quality of care and patient safety; (2) Fostering partnerships and collaborative networks for quality of care; (3) Sharing Member States' healthcare responses during the COVID-19 pandemic; (4) Prioritizing the patient perspective and shared decision-making in healthcare; (5) Advancing the development, implementation, and evaluation of evidence-based national quality of care plans. Such case studies formed the basis of the Frontiers in Health Services Research Topic, “Innovations in Quality of Care”. The aim was to invite authors to submit recent research and policy work related to innovative approaches to solving local, regional, national, and global quality of care challenges.

The work by Benning et al. was conducted in Germany, and its aim was to determine the prevalence of core Ambulatory Care Sensitive Conditions (core-ACSCs) among emergency department patients in an academic tertiary hospital and to evaluate the urgency of these conditions as indicated by triage levels. Additionally, the study aimed to examine factors influencing these admissions to gain an understanding of potentially preventable visits to the emergency department (ED). This study reveals that while core-ACSCs significantly contribute to ED patient volume, they are not the primary factors driving ED crowding. Notably, 65.2% of the patients who presented with what were later confirmed to be core-ACSCs necessitated urgent care. This underscores the role of efficient ambulatory care in preventing ED presentations. Although this warrants further investigation, such findings suggest that these core-ACSCs could be viewed as potentially avoidable ED presentations.

The manuscript by Wang and Liu focused on understanding the diverse demands of older adults in China for home- and community-based integrated care. The findings highlight the complexity of the care needs of the elderly and underscore the importance of designing varied and tailored integrated care sub-models to effectively meet these diverse requirements.

Sener et al. proposed an integrated approach that combines technology-enabled care with continuous monitoring of hospital capacity to improve care and manage capacity during outbreaks. They emphasized the importance of including patients at high risk for severe infectious diseases who cannot be hospitalized. By identifying patients at risk for future deterioration based on evidence-based risk factors, this approach allows prioritization for remote care programs, taking into account real-time hospital capacity. With the availability of home-based antiviral and antibacterial treatments, such remote care programs are essential. They regulate the flow of patients to hospitals during capacity shortages, provide safe and effective treatment at home, and help improve patient safety and quality of care, particularly by reducing hospital length of stay, thus freeing up hospital resources.

The study by You et al. focused on identifying factors that influence depression in older adults living at home. The study found that health status, willingness to help older adults, self-care, and activities of daily living (ADL) significantly influenced geriatric depression. Older adults in the 60–69 years age range had the highest incidence of depression due to changes in physical condition and social roles. The study also found that self-care significantly reduced depression. Higher ADL scores were associated with a lower incidence of depression. This study underscores the importance of improving the quality of care by fostering self-care, social support, and mutual aid among older adults to reduce depression and improve their quality of life.

The manuscript by Chibi et al. provided a comprehensive review of various digital and device-based innovations, categorizing them as digital platform-based, mobile-based, social media-based interventions, and various devices like drones and rapid diagnostic tools. The findings reveal a technological shift towards the use of big data and Artificial Intelligence, suggesting potential improvements in HIV program delivery. The article advocates for African countries to leverage these technologies for enhanced healthcare delivery, highlighting the importance of privacy, security, inclusivity, technical capacity, and policy infrastructure in adopting these innovations. The study underscores the critical role of technological developments in advancing HIV services and calls for strategic implementation in African contexts to optimize health outcomes.

Finally, the study protocol by Hynes et al. outlines a comprehensive, multi-methods approach to understanding care coordination for veterans with complex care needs within the Veterans Health Administration. The protocol is structured around three primary aims: (1) to examine the relationship between care coordination processes and health outcomes; (2) to assess veterans’ perspectives on care integration and patient-centeredness; and (3) to understand providers' perceptions of care coordination tools and processes. The study's novel approach lies in its comprehensive examination of care coordination from multiple perspectives—patients, providers, and the healthcare system. The protocol serves as a blueprint for future research in similar complex healthcare systems, emphasizing the need for multidimensional assessment and evidence-based care coordination strategies.

As advancements redefine the healthcare landscape, they call for meticulous, tailored strategies that can ensure quality, accessibility, and adaptability to evolving care needs. We aim to inspire researchers to rigorously engage with the topic of innovation in healthcare quality.
